# Supplementary material for: Multi‐dataset identification of innovative feature genes and molecular mechanisms in keratoconus
Source: J Cell Mol Med. 2024 Sep 19;28(18):e70079. doi: 10.1111/jcmm.70079 (PMC11412914; doi:10.1111/jcmm.70079)
Supplement: Supplementary file 2 — Material S2. [file JCMM-28-e70079-s002.zip › 7.GSEA analysis/4.MYRF/KEGG_CITRATE_CYCLE_TCA_CYCLE.html]

Details for gene set KEGG\_CITRATE\_CYCLE\_TCA\_CYCLE[GSEA]

|  || Dataset | MYRF.group.cls#h\_versus\_l |
| Phenotype | group.cls#h\_versus\_l |
| Upregulated in class | h |
| GeneSet | KEGG\_CITRATE\_CYCLE\_TCA\_CYCLE |
| Enrichment Score (ES) | 0.22454794 |
| Normalized Enrichment Score (NES) | 0.90238225 |
| Nominal p-value | 0.5508475 |
| FDR q-value | 1.0 |
| FWER p-Value | 1.0 |
Table: GSEA Results Summary

  

Fig 1: Enrichment plot: KEGG\_CITRATE\_CYCLE\_TCA\_CYCLE      
 Profile of the Running ES Score & Positions of GeneSet Members on the Rank Ordered List

  

| SYMBOL | TITLE | RANK IN GENE LIST | RANK METRIC SCORE | RUNNING ES | CORE ENRICHMENT || 1 | OGDHL | na | 2666 | 0.313 | 0.0810 | Yes |
| 2 | SUCLG2 | na | 2914 | 0.299 | 0.2245 | Yes |
| 3 | DLST | na | 12010 | 0.063 | -0.0057 | No |
| 4 | PDHB | na | 12493 | 0.057 | 0.0090 | No |
| 5 | DLD | na | 13282 | 0.046 | 0.0096 | No |
| 6 | SDHC | na | 13938 | 0.039 | 0.0103 | No |
| 7 | FH | na | 14305 | 0.034 | 0.0170 | No |
| 8 | PC | na | 14426 | 0.033 | 0.0303 | No |
| 9 | PDHA2 | na | 15594 | 0.022 | 0.0077 | No |
| 10 | MDH2 | na | 15820 | 0.020 | 0.0110 | No |
| 11 | CS | na | 16971 | 0.009 | -0.0176 | No |
| 12 | SUCLG1 | na | 17093 | 0.008 | -0.0171 | No |
| 13 | SDHD | na | 18021 | -0.001 | -0.0435 | No |
| 14 | ACLY | na | 18106 | -0.001 | -0.0452 | No |
| 15 | IDH3A | na | 19250 | -0.013 | -0.0714 | No |
| 16 | PDHA1 | na | 19670 | -0.018 | -0.0745 | No |
| 17 | MDH1 | na | 19736 | -0.019 | -0.0670 | No |
| 18 | ACO2 | na | 20143 | -0.024 | -0.0668 | No |
| 19 | OGDH | na | 20192 | -0.024 | -0.0560 | No |
| 20 | DLAT | na | 20411 | -0.027 | -0.0489 | No |
| 21 | SDHA | na | 20549 | -0.028 | -0.0387 | No |
| 22 | IDH2 | na | 21549 | -0.040 | -0.0472 | No |
| 23 | IDH3B | na | 22291 | -0.049 | -0.0437 | No |
| 24 | IDH3G | na | 22313 | -0.050 | -0.0193 | No |
| 25 | IDH1 | na | 23974 | -0.075 | -0.0292 | No |
| 26 | SDHB | na | 23984 | -0.075 | 0.0085 | No |
| 27 | ACO1 | na | 24094 | -0.077 | 0.0444 | No |
| 28 | SUCLA2 | na | 24902 | -0.091 | 0.0671 | No |
| 29 | PCK1 | na | 28560 | -0.177 | 0.0508 | No |
| 30 | PCK2 | na | 30749 | -0.252 | 0.1147 | No |
Table: GSEA details [plain text format]

  

Fig 2: KEGG\_CITRATE\_CYCLE\_TCA\_CYCLE      
 Blue-Pink O' Gram in the Space of the Analyzed GeneSet

  

Fig 3: KEGG\_CITRATE\_CYCLE\_TCA\_CYCLE: Random ES distribution      
 Gene set null distribution of ES for **KEGG\_CITRATE\_CYCLE\_TCA\_CYCLE**
